# Unlocking sociocultural and community factors for the global adoption of genomic medicine

**DOI:** 10.1186/s13023-022-02328-3

**Published:** 2022-05-12

**Authors:** Lynsey Chediak, Nicola Bedlington, Ayesha Gadson, Alastair Kent, Aiedah Abdul Khalek, Luke Rosen, Malisa Rust, Mohd. Farooq Shaikh, Meng Yoe Tan, Samuel Agyei Wiafe, Gareth Baynam, Charles A. Steward

**Affiliations:** 1 Patient Advocacy and Engagement Advisory Board, Congenica Ltd, Wellcome Genome Campus, Hinxton, CB10 1DR UK; 2 Rarebase PBC, 1804 Embarcadero Road, Palo Alto, CA 94303 USA; 3 Western Australian Register of Developmental Anomalies, King Edward Memorial Hospital, Perth, WA Australia; 4 Malaysia Immersion Hub, Monash University Malaysia, 47500 Bandar Sunway, Selangor Malaysia; 5 Jeffrey Cheah School of Medicine and Health Sciences, Monash University Malaysia, 47500 Bandar Sunway, Selangor Malaysia; 6 School of Arts and Social Sciences, Monash University Malaysia, 47500 Bandar Sunway, Selangor Malaysia; 7 Rare Disease Ghana Initiative, F393 Fourth Otswe Street, Accra, Ghana; 8 KIF1A.ORG, 808 Columbus Avenue, New York, NY 10025 USA; 9 Rare Care, Clinical Centre of Expertise for Rare and Undiagnosed Diseases, Perth Children’s Hospital, Perth, WA Australia; 10 Congenica Ltd, Wellcome Genome Campus, Hinxton, CB10 1DR UK

**Keywords:** Sociocultural factors, Diagnosis, Equity, Genomic medicine, Genomic sequencing, Rare disease

## Abstract

Advances in genomic sequencing and genetic testing are increasingly transforming the diagnosis and treatment of diseases—specifically, rare diseases. However, the application and benefit of such technologies remain inequitable globally. There is a clear and urgent need to provide genomic sequencing to people across the global population, including people living in under-resourced areas and/or underrepresented populations. Financial considerations are the most obvious barriers to the adoption of genomic medicine, yet there are many other factors that are not so obvious, such as geography, language, communication, and culture. Herein, we use the lens of rare diseases and focus on firstly, selected socio-cultural factors, and in particular stigma; and secondly, empowering community factors such as education, advocacy and connectivity amongst people living with rare diseases globally. These are critical areas of need and opportunity if genomic medicine is to achieve equitable and global adoption in the patient best-interest across low- middle- and high-income country health systems. Furthermore, we touch on specific child health aspects and how they can point towards opportunities to build on specific infrastructures.

## Introduction

Patient and clinician access to genomic sequencing and genetic testing broadly has proven to accelerate the pathway to diagnosis and treatment for devastating diseases—rare, common, acute, and chronic. The advent of genomic sequencing and the uptake of this technology by healthcare systems as a mainstream tool provides hope for the future to patients and families affected by disease across the globe. Yet this technology is predominantly used in high income (HI) countries with their ability to use existing infrastructures (or to create new ones) to enable access to genomic sequencing and the resulting clinical efficacy. This growing field of using such genomic sequencing and the information it provides in a clinical setting is called genomic medicine. Although expanding in its applications, the National Institutes of Health in the United States defines genomic medicine broadly as “an emerging medical discipline that involves using genomic information about an individual as part of their clinical care (e.g. for diagnostic or therapeutic decision-making) and the health outcomes and policy implications of that clinical use” [1]. The expansion of genomic medicine, notably driven by the example of the Genomic Medicine Servic of The National Health Service (NHS) in England, begs the question: how long will it take other countries to similarly offer access to genomic medicine? What key barriers exist to the implementation of genomic medicine?

Given the current disparities in access to genomic medicine, it is evident that the adoption of genomic medicine is globally inequitable. There is an urgent need to provide clinical-grade genomic sequencing to people in need of this resource across the global population—this includes people living in under-resourced areas and/or underrepresented populations. Moreover, this access needs to occur in a way that is clinically efficacious and trusted, allowing for community engagement and a culturally safe atmosphere. There are a host of factors that influence access to genomic medicine, including but not limited to awareness, financial means, health service infrastructure, geography, language, communication, and culture.

Herein, we use the lens of rare diseases and focus on firstly, selected socio-cultural factors, and in particular stigma; and secondly, empowering community factors such as education, advocacy and connectivity amongst people living with rare diseases (PLWRD). These are critical areas of need and opportunity if genomic medicine is to achieve equitable and global adoption in the patient best-interest across both HI countries and low- and middle-income countries (LMICs).

## Rare diseases and genomic medicine adoption in low- and middle-income countries

Despite the presence of the word ‘rare’ it is increasingly evident that rare diseases are prevalent from a global perspective—and, most critically, represent a patient population that is tremendously underserved. Globally, the number of PLWRD is estimated to be between 263 and 446 million [2]. For these millions of people globally living with a rare disease, 80% are suspected to be genetic disease—meaning that although some have known diagnoses, frequently children are living with an unknown, undiagnosed or misdiagnosed disease. It takes an average of 5 years to reach a diagnosis for a child suspected of having a rare disease [3]. If a diagnosis is reached, the disparity widens with 90% of rare genetic diseases offering no available treatment or therapeutic modality [4].

Accurate diagnosis of these complex medical conditions would be faster and more affordable with global access to genomic medicine; without access to genomic sequencing, it is the norm for children living with a rare disease to receive a misdiagnosis or remain undiagnosed for years, if not their entire lifetime. Children in HI countries affected by rare diseases often wait months or years to receive a diagnosis. This painful diagnostic odyssey includes visits to multiple specialists for redundant tests and procedures, which are too often invasive, painful and sometimes require a general anesthetic. Lack of a definitive genetic diagnosis leads to misdiagnosis at the clinical level, resulting in uninformed treatment plans by clinicians forced to work without the genomic tools they need to properly diagnose a patient affected by a rare, genetic disease.

While rare diseases are a prime case study for the effective use of genomic medicine in HI countries, access to diagnosis and thus appropriate treatment is incredibly challenging in LMICs. One community leader and the Founder of the Rare Diseases Ghana Initiative (RDGI), Samuel Wiafe describes: “the only way a child suspected for a rare disease can get a whole genome sequence [in Ghana] is through the RDGI-Illumina iHope Program.” This program, however, only offers such services to 10 patients a year [5]. This initiative highlights critical gaps in the reference genome. If these samples are sent out of the country to be sequenced and compared to the European-centric reference genome, will these resulting diagnoses be accurate?

Similar challenges to access exist across Southeast Asia where a multiplicity of cultures, backgrounds, ethnicity, and lifestyle differences all affect access to genomic sequencing and a genetic diagnosis for a rare disease. The region is lagging in compiling genetic databases, so, even with the cost of genetic testing falling, there are ethical concerns on how the genetic information might be used. The basis of genetic testing or genomic medicine depends on cataloguing local genomes, which is lacking in that region.

The head of SingHealth Duke-NUS Genomic Medicine Centre in Singapore, Dr. Saumya Jamuar said “The lack of local reference data is the biggest challenge for promoting genomic medicine in Asia. The pitfall would be if we continue to ignore this problem and not build that local reference database. Leading nations in precision medicine research, including the U.K., U.S., China, Japan and Saudi Arabia are compiling large genome collections. The U.K. appears to be the front-runner with 100,000 genomes cataloged as of 2018; the country now targets to sequence 5 million genomes by 2024. We want to be in a similar space, and we want to be ready as well."

Thailand is another country which is making progress towards the adoption of genomic medicine, but only after the Thai Government invested in a $150 million 5-year Genomics Thailand Initiative, which aimed to catalog the genomes of 50,000 Thai people [6].

Malaysia is also moving in a similar direction and hopes to sequence around 50,000 genomes in the next 5 years from its own population. The rolling out of a national genome sequencing initiative will test and profile patients with different diseases. Professor Rahman Jamal, a leading geneticist from Malaysia, describes the initiative: “It is crucial that we continue to generate evidence that precision medicine is cost effective and improves the outcome and quality adjusted life years in our patients.” [7]

The ongoing experiences of these communities in Ghana, Singapore, Thailand, and Malaysia are collectively informing how to improve the global clinical adoption of genomic medicine. There are similar challenges to access for Indigenous populations that highlight individual and intersecting access barriers to genomic medicine. The focus for analysis on this important research similarly found that access to genetic services, concerns about data sharing, and lack of education on how to handle biospecimens need to be addressed [8].

Beyond these systematic barriers to genomic medicine in LMICs, there is a lack of literature and research on the systemic socially- driven barriers as they relate to the community’s role and lack of activity in education, advocacy and connectivity. These core issues are explored below.

## Socio-cultural perspectives on rare diseases

The frequently complex multi-system, whole-of-life, whole-of-lifespan and cross-sector needs of PLWRD and their families requires a holistic approach that is broader than biology alone; socio-cultural factors play a critical role in genomic medicine adoption [9].

For instance, religious beliefs can play a critical factor in modifying access to and the conceptualization of a rare disease diagnosis. Many religions believe that egregious acts in past life result in current suffering​ or that all illnesses, whether physical or mental, have a biological, psychological and spiritual element. Existing approaches which do not address and resonate with underlying religious beliefs may have limited adoption and efficacy. Illness, suffering, pain and dying may be seen as a test from God, so illness becomes a trial by which one's sins are removed. Often, health is placed second in importance to faith. In some cultures, without citing a single specific one as to avoid further objectification, responses to genetic conditions may also be influenced by belief-systems which stress a range of beliefs such as: absolute obedience to one's parents and to adults in general or illness resulting from forces such as “spirit intrusion,” “violation of taboos,” “soul-loss,” or “disease sorcery”. For these reasons, rare genetic diseases may produce shame and social stigma with consequences that have been reported in literature on Asian cultural views of genetic services. Furthermore, we have also observed that in certain communities and cultures, there is a strong reluctance to seek a genetic diagnosis. For example, a genetic diagnosis can materially affect PLWRD by implicating their family’s prospects in terms of marriage,  wealth and/or  wellbeing. Subsequently, this can prevent families from seeking such a diagnosis [10].

In Baker’s work in epilepsy as an example, he identifies that culture, demographic, illness-related and psychosocial factors can all predict stigma to varying degrees [11]. Higher levels of perceived stigma are associated with a reduced sense of self-efficacy, poor epilepsy outcomes and seizure severity. Frequently identified predictors of enacted stigma include low level of knowledge about epilepsy, lower educational level, social class and socioeconomic status, living in a rural area, and religious grouping [11]. Variation in stigma across geographical regions and within regions and countries is also significant in epilepsy. Regions with a strong cultural perception of disease that rely on non-scientific explanations of epilepsy, such as causes rooting from spiritual, contagion or forms of insanity tend to have poorer attitudes towards people living with epilepsy [12].

The importance of identifying socio-cultural barriers to diagnosis of rare diseases and genomic medicine is crucial. Through this process of navigating the complexities of specific local cultures, it is possible to identify spheres of influence, including the individuals and institutions who are gatekeepers of information and command the trust of their respective communities. Engaging with the right gatekeepers is a possible way to communicate relevant information to these communities. To use the prior example of stigma in religious contexts, it is possible that trust in religious leaders might be higher than trust in other individuals. As such, equipping these leaders with the relevant information for dissemination of information may assist in circumventing medical skepticism.

While the stigma associated with a rare genetic diagnosis is not confined to LMICs, the proportion of burden associated with it may be, overall, greater than in HI countries.

## The fundamental role of education, advocacy and connecting with other people

For most families, what genomic sequencing might reveal remains a mystery. There is still, in many cases, a sense of absolute genetic determinism which leads to false expectations about the likelihood of getting a result from a genetic test and its significance. There is sometimes a lack of understanding that a medical condition can result from one single change in a gene—rather it is assumed that purely environmental changes may be to blame. Therefore, all communication by those providing and using genomic analyses must be comprehensive enough to give families an insight into the specific rare disease they are facing and the possible root cause.

Education about genomic medicine is essential: both the benefits and the limitations of what is currently possible from the perspective of patients, clinicians and scientists. Information on rare genetic diseases is constantly expanding, but incomplete. However, accurate information is still beneficial to no information at all. Given the reality that many clinicians and/or scientists work in isolation on specific rare disease areas, they would benefit hugely from being in front of patients who can share their story and lived experiences. While we should be cautious, we should also be excited about the future for better connecting patients affected by the same disease.

Learning about the genetic cause for an individual’s condition in the form of a definitive diagnosis is a key step in clinical management, but also on a patient’s educational journey to understand what is happening to their body and to be able to connect with the sparse population of people affected by the same disease.

In the case of a child receiving a diagnosis for a rare genetic disease, it also provides a disease label and typically relieves the parental grief that can occur amid a long-lasting diagnostic odyssey; as a result, the parents of children affected by rare disease represent a dominant voice driving advocacy of behalf of their children. Patients and families are, ultimately, the most inspirational and empowered group of people, which can be seen from the numerous condition-specific patient-led support groups and charities, both large and small.

Large multi-stakeholder and advocacy groups are emerging both nationally and internationally. For example, EURORDIS, which stands for The European Organization for Rare Diseases, represents 988 different rare disease patient organizations from 74 different countries who are all working together to strengthen the patient voice in order to shape research, policies, and patient services [13]. Similarly, in the United States, the National Organization for Rare Disorders (NORD) represents more than 300 different rare disease patient organization members nationally and leads programs on education, advocacy, research, and patient services and Global Genes exists to connect people living with rare diseases [14, 15]. These types of organizations are also present in other HI countries such as the Canadian Organization for Rare Disorders (CORD), the Chinese Organization for Rare Disorders, Rare Voices Australia (RVA), and the Genetic Alliance UK. Internationally, Rare Diseases International (RDI) brings regional and national rare disease patient organisations together. The International Rare Diseases Research Consortium (IRDiRC) is a global collaborative multi-stakeholder initiative launched in 2011 by both the European Commission and the U.S. National Institutes of Health “to enable all people living with a rare disease to receive an accurate diagnosis, care, and available therapy within one year of coming to medical attention” [16]. It has active advocacy participation and leadership, including through the Patient Advocates Constituent Committee which includes representatives of national or supra-national advocacy organizations. Collectively, these many organizations and the subsidiary patient-led organizations they represent, have worked to connect patients facing the same disease and to bring attention to the burden of rare diseases at an international level, such as with the United Nations Resolution adopted on December 16, 2021 ‘Addressing the Challenges of Persons Living with a Rare Disease and their Families’ [9].

While advocacy groups are progressing in LMICs, as exemplified by the RDGI, the Indian Organization for Rare Disorders (IORD) or the Group of Alliance, Investigation and Support in Rare Diseases (GEISER) and ERCAL, which are working to connect people across Latin America and the Caribbean with rare diseases, voices from these countries and other similar communities are often lacking.

Where it is possible to get an accurate diagnosis thanks to genomic sequencing, many disease-specific patient-led organizations are forming in HI countries allowing for patients to connect, track their symptoms, and in some cases, fund a treatment for their rare disease. For instance, the Cystic Fibrosis Foundation in the U.S. funded the development of the first approved drug to address the underlying cause of cystic fibrosis called ivacaftor (Kalydeco) in 2012 [17].

Ultimately, advocacy and community groups allow for a greater understanding of how each rare disease manifests in daily life and may help identify possible ways to reduce disease progression outside of a clinical setting. By joining together, PLWRD and their families can push to make their respective health system more effective while also driving progress in research and diagnostics. If such communities are to be enabled to benefit from the potential of genomic testing for rare diseases, then the barriers to their formation need to be understood and factored into the planning and delivery of health care. PLWRD globally must come together to explore our understanding and the perceptions of genomics in their specific region and/or cultural existence.

## A focus on children and youth

The majority of rare diseases start exclusively in children (7 in 10) or have their onset in either childhood or adulthood (an additional 2 in 10) [2]. Moreover, there is a greater proportion of children and youth populations in LMICs when compared to HI countries. For instance, 40% of the African population is aged 15 years or younger [18]. Additionally, globally 6% of children are born with one or more birth defects (also called congenital anomalies) [19], which is one of the leading causes of death in newborns and infants—in addition to being a leading cause of suffering and stigmatization known to impede the diagnosis and care of children with birth defects [20]. Birth defects are a large class of mainly rare diseases and the largest class of rare diseases in children [21]. The causal overlap between rare diseases and birth defects (monogenic, environmental and multifactorial) is substantial. Similarly, there are large overlaps in socio-cultural and other access factors. Investigating these synergies, and building on existing infrastructures, between rare diseases and birth defects provide opportunities for global genomic medicine adoption. Some countries have focused genomic medicine initiatives to include childhood cohorts from their outset (e.g. Genomics England), while others (e.g. China) have identified this as a fundamental area that requires increased focus that has been unmasked through initial national initiatives [22].

## Conclusion

From the discussion above, it is clear that while much of the focus on barriers to genomic medicine adoption is on health systems and financial constraints, an increased focus on sociocultural and community factors is critical for delivering insights for a broader and more global implementation of genomic medicine. As many HI countries are expanding the use of genetic testing for PLWRD, LMICs have an opportunity to capitalize on these foundations and deliver greater equity and efficacy by taking a community-first approach that is tailored to the local context, including important cultural factors. To maximize and sustain  improvements in the quality of life of PLWRD globally with  genomic medicine, addressing socio-cultural differences and stigmas is of paramount importance.

## Data Availability

Not applicable.

## Abbreviations

CORD: Canadian Organization for Rare Disorders
EURORDIS: European Organization for Rare Diseases
GEISER: Group of Alliance, Investigation and Support in Rare Diseases
HI: High income
IORD: Indian Organization for Rare Disorders
IRDiRC: The International Rare Diseases Research Consortium
LMICs: Low- and middle-income countries
NORD: National Organization for Rare Disorders
PLWRD: People living with rare diseases
RDGI: Rare Diseases Ghana Initiative
RDI: Rare Diseases International
RVA: Rare Voices Australia
